# Silicon application enhances drought resilience in buckwheat: a comparative study of three varieties

**DOI:** 10.3389/fpls.2025.1635709

**Published:** 2025-09-23

**Authors:** Jiri Krucky, Vaclav Hejnak, Pavla Vachova, Aayushi Gupta, Jan Kubes, Marek Popov, Milan Skalicky

**Affiliations:** Department of Botany and Plant Physiology, Faculty of Agrobiology, Food and Natural Resources, Czech University of Life Sciences Prague, Prague, Czechia

**Keywords:** *Fagopyrum esculentum*, RWC, proline, malondialdehyde, 5-methylcytosine, osmotic potential, leaf gas exchange

## Abstract

This study evaluated the effects of foliar silicon (Si) application on physiological and biochemical traits in three buckwheat lines (La Harpe, Panda, Smuga) grown under optimal (control) and drought stress conditions. Plants were cultivated under controlled conditions with four treatments: Control (80% water availability), Drought (40%), Control + Si, and Drought + Si (0.5 mM Na_2_SiO_3_·9H_2_O applied to foliage). Water stress significantly reduced relative leaf water content (RWC), osmotic potential (Ψ_s_), photosynthetic pigments, and gas exchange parameters (*A*, *E*, *g_s_*) in all varieties. It also increased malondialdehyde (MDA), total flavonoid content (TFC), total antioxidant capacity (TAC), and 5-methylcytosine (5mC), while Fv/Fm remained unchanged, indicating sustained photosystem II activity. However, varietal differences were evident. La Harpe and Panda showed lower RWC, Ψ_s_, *A*, *E*, and *g_s_* under drought than Smuga. La Harpe had the highest MDA accumulation in roots, increased 5mC levels in leaves, and was the only line with decreased water use efficiency (WUE). Smuga exhibited the highest natural proline level and the strongest proline increase under drought. Foliar Si application reduced MDA and enhanced antioxidant activity (TFC, TAC) in both roots and leaves across all varieties, under both water regimes. The strongest antioxidant response was observed in La Harpe. Si also improved photosynthetic pigment levels, likely contributing to the protection of the photosynthetic apparatus under drought stress. Its effects on RWC, Ψ_s_, and gas exchange under drought were variety-specific: La Harpe and Panda responded positively, while Smuga showed minimal changes. Group correlation analysis under drought showed that Smuga had the strongest positive correlations between plant health traits and stress responses, suggesting effective physiological coordination. Panda showed moderate, and La Harpe negative, correlations. After Si application, these relationships improved most in Smuga, moderately in Panda, and least in La Harpe. Overall, the results reveal clear genotype-specific responses to foliar-applied Si in buckwheat. Silicon improved antioxidant defenses, mitigated drought-induced oxidative stress, and supported physiological functions, particularly in Smuga. These findings support using Si as a promising tool to enhance drought resilience in buckwheat cultivation.

## 1 Introduction

Drought is one of the most serious abiotic stressors that negatively affects agricultural crop growth, development and production (Alam et al., 2024; Hossain et al., 2024). In addition to reducing photosynthetic activity, nutrient uptake and distribution, a plant’s water deficit can increase the accumulation of reactive oxygen species (ROS) and the development of oxidative stress (Akhtar and Ilyas, 2022; Ahsan et al., 2023). In recent years, there has been increasing interest in the use of silicon (Si) as a potential plant biostimulant to mitigate the negative effects of abiotic stress factors, especially drought (Sharf-Eldin et al., 2023; Zahedi et al., 2023). Foliar application of silicon compounds has been shown to be an effective way of supplying the element to crops (Ahsan et al., 2023).

Applying Si to plants subject to water deficits can activate specific defense mechanisms. Silicon has been shown to enhance chlorophyll content (Zhang et al., 2018; Ahsan et al., 2023) and reduce stomatal conductance and transpiration, thereby contributing to the maintenance of efficient photosynthesis (Al-Selwey et al., 2023; Nazim et al., 2024; Sattar et al., 2023; Sharf-Eldin et al., 2023). Si can also stabilize a plant’s water balance by improving the osmotic potential (Ψ_s_) in leaves (Saja-Garbarz et al., 2021; 2024) and maintaining a higher relative water content (RWC) (Desoky et al., 2021; Ning et al., 2023).

In response to drought, plants often accumulate the amino acid proline, which plays a key role in osmoregulation, protection of cellular structures, and free radical elimination (Akhtar and Ilyas, 2022; Morshedloo et al., 2025). In addition, Si can reduce the level of malondialdehyde (MDA), a marker of lipid peroxidation and oxidative stress (Mahmoud et al., 2023; Zadegan et al., 2023) by stimulating flavonoid synthesis (TFC) and antioxidant defense systems, which increases total antioxidant capacity (TAC) (Desoky et al., 2021; Handaragamage and Abeysinghe, 2024; Morshedloo et al., 2025). Silicon’s mechanism of action may also involve epigenetic regulation, e.g., by altering the levels of 5-methylcytosine (5mC), which plays a role in the plant’s response to stress (Rao et al., 2024; Zi et al., 2024).

Common buckwheat (*Fagopyrum esculentum* Moench.) is one of the pseudocereals with increased sensitivity to drought (Aubert et al., 2021; Germ et al., 2025). Although studies investigating the effect of drought on different aspects of buckwheat physiology are available (Oksana et al., 2023; Rangappa et al., 2023; Hossain et al., 2024), a detailed understanding of the effects of foliar application of silicon on physiological and biochemical parameters in different buckwheat lines under drought conditions is still lacking.

This study aims to elucidate the mechanisms by which foliar application of silicon affects physiological (RWC, Ψ_s_, Fv/Fm, *A*, *E*, *g_s_*, WUE, WUE_i_), biochemical (proline content, MDA, TFC, TAC, chlorophyll *a*, chlorophyll *b*, total chlorophyll and carotenoids) and potentially epigenetic (5mC) parameters in selected buckwheat cultivars under stress from water deficiency. We anticipate that the results will indicate which buckwheat variety has the highest drought resistance and extend our knowledge about the physiological mechanism of the effect. Our goal is to mitigate the negative effects of dry periods due to climate change by optimizing agronomic strategies for silicon application.

## 2 Materials and methods

### 2.1 Plant material and experimental conditions

The common buckwheat (*Fagopyrum esculentum* Moench.) cultivars, La Harpe (French origin), Panda (Polish origin), and Smuga (Polish origin) were selected as experimental subjects. These cultivars were chosen based on contrasting responses to drought stress observed in preliminary testing, conducted in cooperation with Poznań University of Life Sciences under a bilateral Czech–Polish research project. These three cultivars were selected based on their contrasting physiological responses to water limitation and agronomic traits, making them suitable for comparative analysis of silicon (Si)-mediated drought mitigation.

La Harpe is a French cultivar included in the official variety list of France and used in the production of buckwheat flour under the protected geographical indication “Farine de blé noir de Bretagne.” It is considered a moderately late-flowering variety, typically grown in Western Europe, with good adaptability to temperate conditions and a relatively stable seed set. It also contains favorable levels of flavonoids and low husk content (Girerd, France).

Panda is a well-established Polish cultivar characterized by mid-early maturity and good adaptation to continental and moderately cold conditions. It is cultivated primarily for food-grade uses and is suitable for low-input farming systems (PH “SIM” Sławomir Herman, Poland).

Smuga is another improved Polish cultivar known for high yield potential (up to 2.7 t/ha), enhanced lodging resistance, and greater drought tolerance. It exhibits a higher 1000-seed weight and protein content compared to other available Polish varieties (Logistic Sp. z o.o., Poland).

Seeds of selected varieties were sterilized by soaking in 1% NaClO for 5 min and then rinsed several times with distilled water. The seeds were then allowed to dry on a paper towel to achieve natural moisture. The sterilized seeds were sown in plastic pots (11 × 11 × 23 cm) filled with peat substrate (Klasmann TS2, Germany). The pot experiment was carried out in a plant growth chamber (Conviron E8, Winnipeg, Canada) with a CMP6050 control system at an artificial light intensity of 750 µmol m^−2^ s^−1^, a photoperiod of 14/10 h (day/night), a temperature of 23°C/18°C (day/night) and relative humidity of 50%/60% (day/night).

### 2.2 Experimental setup and treatments

The cultivation phase lasted 18 days, during which all the plants were regularly irrigated until the 3rd to 5th true leaves appeared. This was followed by the experimental phase, where the pots (five for each variety with four plants per pot) were randomly divided into four treatments: Control (irrigated, without Si), Drought (dry, without Si), Control + Si (irrigated, with 0.5 mM Si), Drought + Si (dry, with 0.5 mM Si). The silicon formulation (Na_2_SiO_3_·9H_2_O; Merck KGaA, Darmstadt, Germany; Si) was dissolved in distilled water at the above concentration and sprayed evenly on the leaves to ensure complete leaf coverage, once at the beginning of the experimental phase using a hand-held pressurized sprayer (SOLO 402, Kleinmotoren GmbH, Germany). Before contact with the silicon formulation, the substrate was carefully covered with aluminum foil, which was removed from the pots after the solution had dried on the leaves.

Regular gravimetric measurements monitored the operation of the watering system. Every two days, the pot water was checked and maintained at 80% of the substrate water capacity to reach the optimum hydration level in the irrigated groups (Control, Control + Si). Drought stress was induced by limiting watering, which gradually reduced the moisture level to 40% of the substrate water capacity in the stressed variants (Drought, Drought + Si), and this level was maintained throughout the experimental phase (12 days). At the end of the drought period, plant material was sampled, and the studied parameters were measured. All data values are based on five replicates.

### 2.3 Relative water content of leaves

The gravimetric method determined relative water content (RWC) on fully expanded leaves. In this method, five identical targets were taken from the center of the leaf and weighed for their fresh weight (FW), the turgor weight (TW) of these segments after saturation with water for 4 h, and the dry weight (DW) of these segments after drying in an oven at 90 °C for 3 h. The RWC was then calculated by the formula below:


$$RWC\;\left(\%\right)=\frac{\;\left[FW-DW\right]}{\left[TW-DW\right]}\;\times \;100$$

### 2.4 Leaf osmotic potential

Leaf osmotic potential (Ψ_s_) was determined using a Psypro instrument (Wescor Inc., Logan, Utah, USA). First, buckwheat leaf samples were collected and placed in 5 ml syringes, sealed with Parafilm and frozen at -24°C. Before measurement, the samples were kept at laboratory temperature until the plant tissues were completely thawed. Subsequently, the contents of the syringe was squeezed into an Eppendorf microfuge tube (1.5 ml), the extract was mixed and 20 µL was pipetted onto a 6 mm diameter sampling disc (ELITech Group Biomedical Systems, Logan, Utah, USA), which was placed into a C-52 sampling chamber (Wescor Inc., Logan, Utah) and the osmotic potential of the leaves was determined in MPa after 45 min of sample stabilization.

### 2.5 Leaf proline content

The method of Bates et al. (1973) with modifications was employed to determine the proline content. A leaf sample (0.5 g) was ground in 10 ml of 3% sulphosalicylic acid using a mortar and pestle, and the suspension was filtered through filter paper. Subsequently, 1 ml of the filtrate was mixed with 1 ml of acidic ninhydrin solution and 1 ml of glacial acetic acid and placed on a shaker (GFL 3005, Verkon Ltd., Prague, Czech Republic) for 15 min. The samples were then heated in a water bath (Memmert WTB, Verkon Ltd., Prague, Czech Republic) at 90°C for 30 minutes, and after cooling, 3 ml of toluene was added to each sample and the tubes were placed on a shaker for 30 minutes. The samples were held for 24 h at 6°C, after which the absorbance at 520 nm of the upper layer of the separated mixture was measured with a spectrophotometer (UV-Vis, Evolution 201, Thermo Scientific, USA). The concentration of proline in µmol g^-1^ fresh weight was determined using a calibration curve.

### 2.6 Malondialdehyde (MDA) content

MDA content in plant tissues was determined using an assay developed by Du and Bramlage (1992). Leaves or roots samples of buckwheat (0.5 g) were ground to a fine powder in liquid nitrogen and suspended in 80% ethanol. The suspension was filtered through Whatman filter paper with 0.45 μm pore size, and the 0.7 ml of reaction solution (consisting of trichloroacetic acid, 2-thiobarbituric acid and 1% butylated hydroxytoluene) was added to the same volume of the filtrate. The reaction mixture was heated in a water bath at 95°C for 20 minutes, and after cooling, the samples were centrifuged at 12,000 rcf (relative centrifugation force) at 4°C for 1 min. The absorbance of the supernatants was measured at 440, 532 and 600 nm using water as a blank in a spectrophotometer (UV-Vis, Evolution 201, Thermo Scientific, USA). The MDA concentration was calculated according to Du and Bramlage (1992) and expressed as nmol g^-1^ leaf fresh weight (FW).

### 2.7 Total flavonoid content (TFC)

The TFC was measured using the method described by Tsanova-Savova et al. (2018). An aliquot of the ethanolic extract from the MDA assay was mixed with 5% NaNO_2_, 10% AlCl_3_, and 1 M NaOH. The solution was immediately measured against a blank at 415 nm at lab temperature, and TFC was calculated as quercetin equivalents (QEs) in mg g^-1^ FW.

### 2.8 Total antioxidant capacity (TAC)

The method for measuring TAC was adapted from Prieto et al. (1999). Aliquots of the same ethanolic extracts from previous assays were mixed with a 1 ml reagent solution containing 0.6 M H_2_SO_4_, 28 mM Na_3_PO_4_ and 4 mM (NH_4_)_6_Mo_7_O_24_. The mixture was heated at 95°C for 90 min, and the absorbance was read at 695 nm against a blank. The TAC was calculated as ascorbic acid equivalents (AAEs) in mg g^-1^ FW.

### 2.9 DNA isolation and 5-methylcytosine determination

Samples of buckwheat leaves were ground in liquid nitrogen with a mortar and pestle. DNA was isolated from samples of plant material (100 mg fresh weight) with a NucleoSpin Plant II isolation kit (Macherey-Nagel GmbH & Co. KG, Dueren, Germany) using the recommended miniprep protocol with PL1 lysis buffer. Global DNA methylation levels (100 ng of DNA) were determined using a fluorometric MethylFlash methylated DNA quantification kit (Epigentek Group Inc., Farmingdale, NY, USA) following the manufacturer’s protocol. Fluorescence (530EX/590EM) was measured on a fluorescence microplate reader (Tecan Infinity M200, Tecan Deutschland GmbH, Crailsheim, Germany) and quantified with Magellan software.

### 2.10 Pigment content

To calculate the concentration of chlorophylls and carotenoids, identical targets were cut from buckwheat leaves using a cork borer and extracted in 1 ml of N, N-dimethylformamide for four hours in the dark. Subsequently, the solutions were placed on a shaker (GFL 3005) for 45 min. Then the absorbance was measured using a spectrophotometer (UV-Vis, Evolution 201, Thermo Scientific, USA) at wavelengths of 663.8 nm, 646.8 nm and 480 nm. The following formulas were used to calculate chlorophyll *a* (Chl *a*), chlorophyll *b* (Chl *b*), total chlorophyll and carotenoids.


Chla=12.0A663.8−3.11A646.8

Chlb=20.78A646.8−4.88A663.8

Total chlorophyll=7.12A663.8+17.67A646.8

(Porra et al., 1989)


Carotenoids=(1000A480−1.12Chla−34.07Chlb)/245

(Wellburn, 1994)

### 2.11 Maximum quantum efficiency of PSII (Fv/Fm)

The FluorPen FP110 portable fluorometer (PSI, Drásov, Czech Republic) was used to evaluate the maximum quantum efficiency of PSII (Fv/Fm). Fully expanded buckwheat leaves were dark-adapted for 20 min using removable leaf clips, and subsequent measurements were performed in the morning between 9 and 11 am.

### 2.12 Leaf gas exchange parameters

The net photosynthetic rate, *A* (μmol CO_2_ m^-2^ s^-1^), the transpiration rate, *E* (mmol H_2_O m^-2^ s^-1^), and the stomatal conductance, *g_s_* (mol H_2_O m^-2^ s^-1^), were measured in fully expanded leaves using the LCpro+ portable gas exchange system (ADC BioScientific Ltd., Hoddesdon, UK). Gas exchange parameters were measured in the morning (between 9 and 11 am) under steady-state conditions in a measurement chamber at 23°C with photosynthetically active radiation (PAR) irradiance at 750 µmol m^−2^ s^−1^. The instantaneous water use efficiency (WUE) was calculated as the ratio *A*/*E*, and the intrinsic water use efficiency (WUE_i_) as the ratio *A*/*g_s_*.

### 2.13 Statistical analysis

All statistical analyses were performed in R (version 4.3.0; R Core Team, 2023) and Microsoft Excel. The analytical workflow included data transformation, descriptive statistics, non-parametric testing, and trait correlation analysis.

To assess treatment effects, we applied the Kruskal-Wallis rank-sum test (Kruskal and Wallis, 1952), a non-parametric alternative to ANOVA suitable for comparing more than two independent groups. Where significant differences were detected, Dunn’s test with multiple testing correction was used for *post-hoc* comparisons.

For each combination of variety and treatment, we calculated medians and interquartile ranges (IQRs) for all physiological and biochemical parameters. These robust summary statistics are appropriate for skewed or non-normally distributed data (Wilcox, 2012). Median values were visualized using bar plots, with IQRs shown as error bars and raw data points overlaid to illustrate within-group variability (Weissgerber et al., 2015). Summary tables of descriptive statistics were compiled for all variables across treatment groups.

We conducted a grouped correlation analysis to evaluate systemic coordination between physiological performance and defense mechanisms. Traits were categorized into health-related and defense-related groups based on their biological roles (as defined in **Supplementary Table S1**). We calculated the mean Pearson correlation coefficient for each variety and treatment across all pairwise combinations between traits from the two groups. This yielded a single summary value — the health– defense correlation — representing the degree of physiological-functional integration under each condition.

To quantify the effect of silicon application under drought, we calculated the difference in health– defense correlation between the Drought + Si and Drought treatments for each variety (denoted as Δ health–defense). This metric served as an indicator of how silicon influenced the coordination between stress response and physiological functioning.

## 3 Results and discussion

### 3.1 Effect of drought and Si treatment on relative water content

RWC is a key indicator of a plant’s hydration and its ability to retain water. Under drought conditions, RWC decreases, which reduces cellular function and can lead to metabolic disturbances and growth retardation. In our experiments, all three buckwheat cultivars showed significant reductions in RWC under drought conditions compared to the Control (**Table 1**). This trend mirrored the results of other studies (Desoky et al., 2021; Condorelli et al., 2022; Ning et al., 2023; Sharf-Eldin et al., 2023). It should be noted that the La Harpe and Panda buckwheat varieties showed significantly lower RWC values under drought stress than the Smuga variety, indicating their lower tolerance to water deficits.

**Table 1:** Effect of foliar application of silicon on relative water content and leaf osmotic potential of different buckwheat varieties under drought conditions.

Variety	Treatment	*Relative water content (%)*	*Leaf osmotic potential (MPa)*
La Harpe	Control	85.52 ± 3.97^c^	-0.85 ± 0.12^a^
	Drought	66.51 ± 0.80^a^	-1.82 ± 0.08^c^
	Control + Si	86.82 ± 3.53^c^	-0.76 ± 0.05^a^
	Drought + Si	72.03 ± 1.59^b^	-1.41 ± 0.10^b^
Panda	Control	86.11 ± 3.17^c^	-0.83 ± 0.09^a^
	Drought	66.41 ± 1.19^a^	-1.89 ± 0.07^c^
	Control + Si	85.54 ± 3.07^c^	-0.83 ± 0.06^a^
	Drought + Si	70.11 ± 0.49^b^	-1.57 ± 0.05^b^
Smuga	Control	86.58 ± 3.41^c^	-0.76 ± 0.06^a^
	Drought	69.36 ± 0.72^b^	-1.63 ± 0.10^b^
	Control + Si	84.78 ± 1.35^c^	-0.85 ± 0.09^a^
	Drought + Si	69.88 ± 0.26^b^	-1.53 ± 0.09^b^
Kruskal-Wallis ANOVA		51.05	51,91
*p*		<0.001	<0.001

Different letters indicate statistically significant differences among treatments (α = 0.05).

We also investigated whether foliar silicon (Si) applications could increase RWC in drought-stressed plants. In the La Harpe and Panda groups, Si application significantly increased RWC compared to untreated plants, but the values were still lower than those of the Control. This increase could be explained by Si’s ability to form a water-retaining silica hydrogel (SiO_2_·nH_2_O) in the leaves, which could raise the RWC by binding free water. Similar results have been reported in wheat and rapeseed (Ning et al., 2023; Sattar et al., 2023; Saja-Garbarz et al., 2024). However, the application of Si did not significantly improve RWC in the Smuga buckwheat variety, which maintained higher RWC values than the other varieties even under drought. This suggests that Smuga naturally possesses different physiological water management mechanisms that confer greater drought resistance. Similar genotypic differences have been described in wheat (Thorne et al., 2021). The application of Si to control unstressed plants did not significantly affect RWC.

### 3.2 Leaf osmotic potential, Ψ_s_

Leaf osmotic potential (Ψ_s_) is an important parameter of plants’ osmotic regulation and water management. Our data showed a strong positive correlation between RWC and Ψ_s_ in all three varieties (La Harpe, r = 0.966; Panda, r = 0.973; Smuga, r = 0.968). In agreement with the literature (Bashir et al., 2021; Condorelli et al., 2022; Guizani et al., 2023), Ψ_s_ values were significantly lower in the three buckwheat cultivars under water deficit compared with regular irrigation in the Control.

Applying Si to drought-stressed plants increased the Ψ_s_ for all plants, but only significantly for La Harpe and Panda (**Table 1**). These results confirm that Si can improve osmotic balance and reduce water loss by transpiration in the more sensitive buckwheat varieties. However, in Smuga, the response to Si application remained statistically inconclusive. This buckwheat maintained a higher Ψ_s_ without Si treatment, possibly because its genetic makeup predisposed it to osmotic adaptation (Aranda et al., 2021; Condorelli et al., 2022).

### 3.3 Proline content

**Figure 1:**
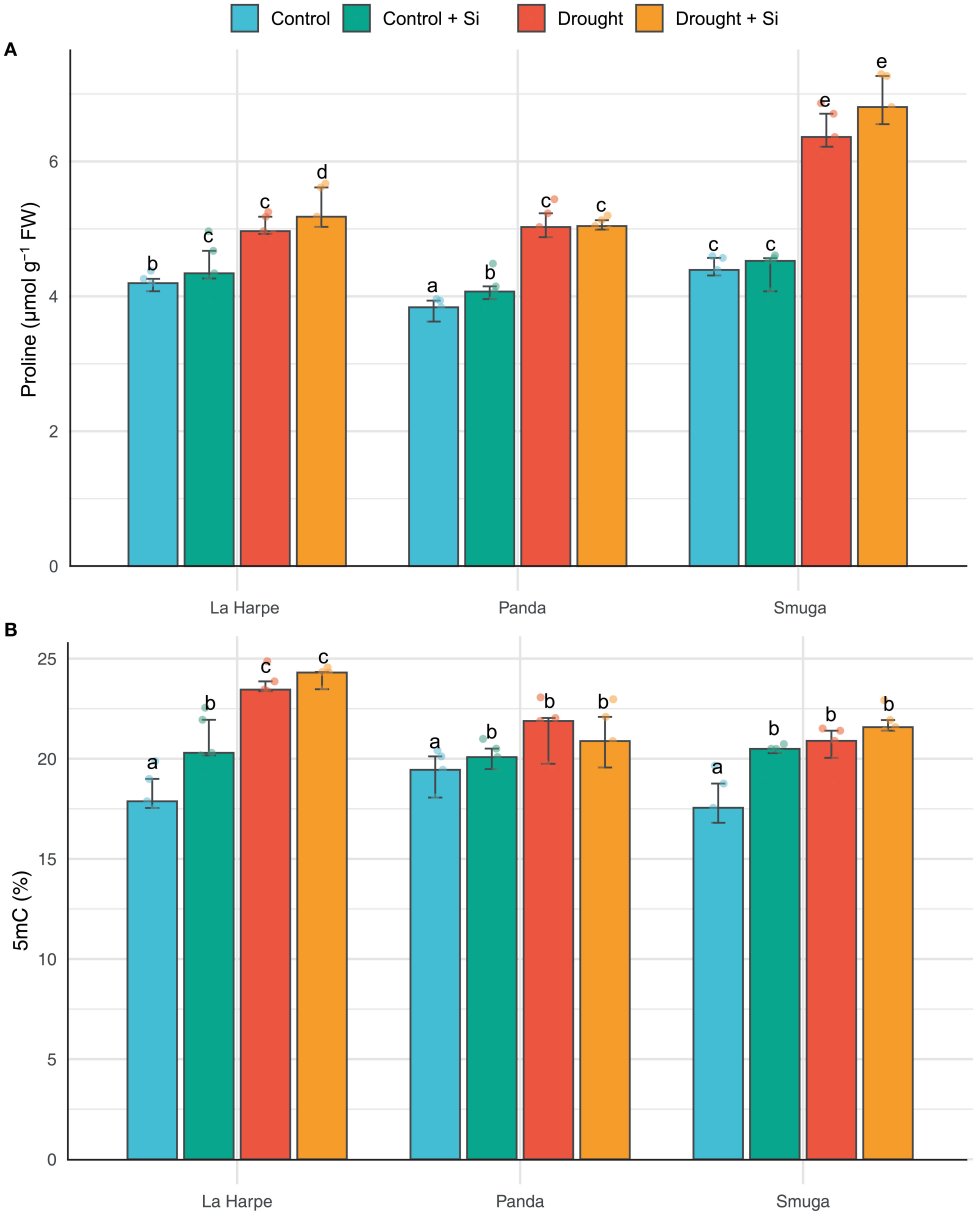
Effect of foliar application of silicon on proline content **(A)** and 5-methylcytosine levels **(B)** of different buckwheat varieties under drought conditions. Data are presented as medians ± IQR. Kruskal-Wallis ANOVA: H = 51.71; *P*< 0.001. Different letters indicate statistically significant differences among treatments.

The proline concentration (µmol g^-1^ FW) is an important indicator of plant response to abiotic stresses. Proline helps to maintain cell turgor, stabilizes proteins and membranes, and acts as an antioxidant (Blum et al., 1999; Chen and Murata, 2002). Our study found significant differences in proline levels between buckwheat varieties under normal irrigation (Controls), with the highest content recorded in Smuga plants. Water deficit stress (both Drought and Drought + Si groups) led to a significant increase in proline accumulation in all varieties (**Figure 1A**), with Smuga showing the greatest proline increase because of its higher level of drought tolerance. These results agree with findings from studies of other crops (Maghsoudi et al., 2018; Mehta et al., 2023; Al-Selwey et al., 2023). A significant increase in proline content was also observed in the Control + Si groups, especially in La Harpe and Panda (**Figure 1A**). This may be related to their naturally lower proline levels and greater responsiveness to Si application (Esmaili et al., 2021; Desoky et al., 2021).

### 3.4 Malondialdehyde content

**Figure 2:**
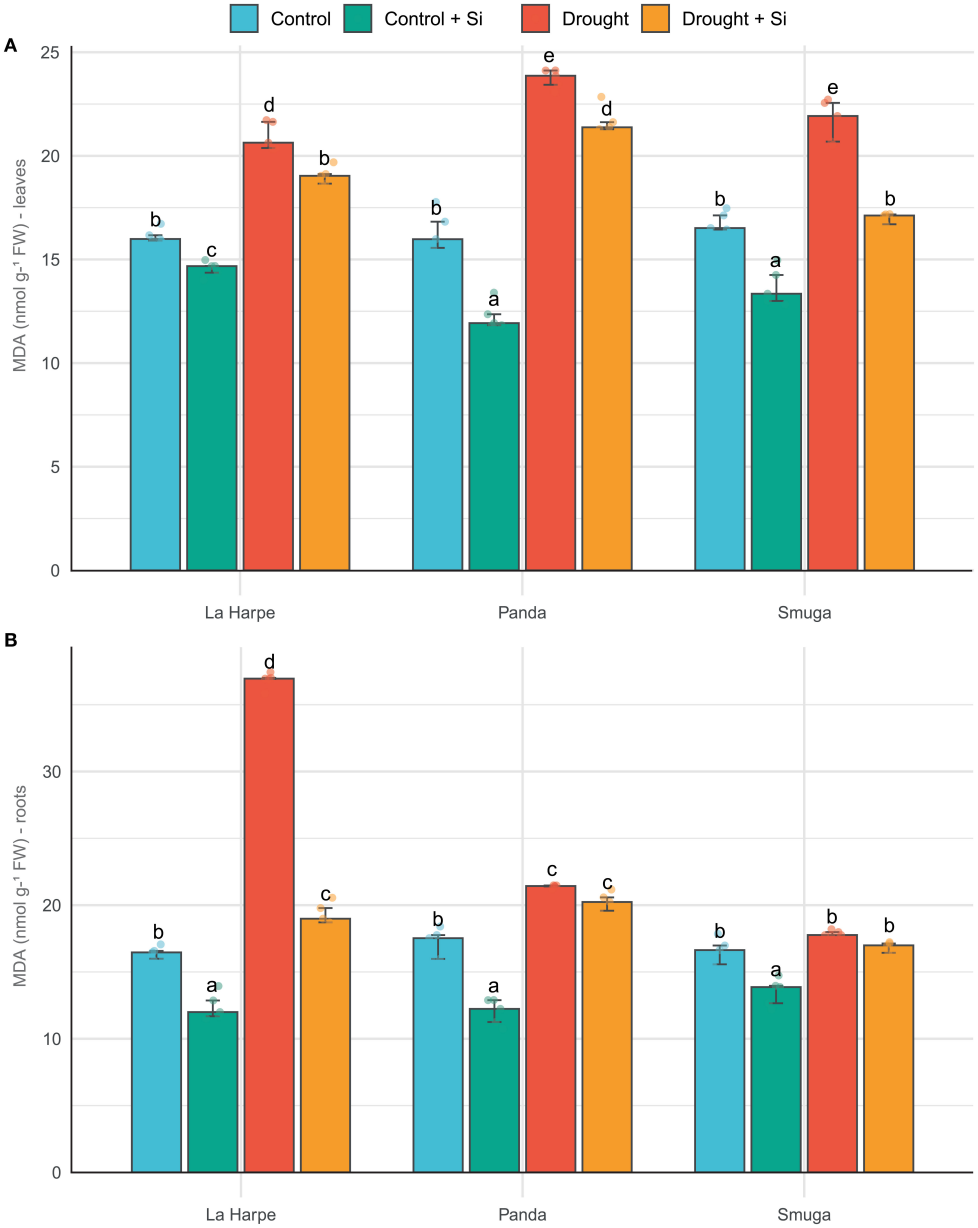
Effect of foliar application of silicon on malondialdehyde content of leaves **(A)** and roots **(B)** of different buckwheat varieties under drought conditions. Data are presented as medians ± IQR. Kruskal-Wallis ANOVA: leaves H = 55.98; *P*< 0.001; roots H = 55.4; *P*< 0.001. Different letters indicate statistically significant differences among treatments.

MDA concentration in plant tissues reflects the level of oxidative stress and the extent of membrane damage by lipid peroxidation resulting from increased production of reactive oxygen species (ROS). In our buckwheat varieties under drought conditions, the MDA content increased in all water-deficient plants compared with controls (**Figure 2**), with the highest increase observed in the La Harpe roots. These results are consistent with findings in different buckwheat species (Aubert et al., 2021; Wu et al., 2019; Hossain et al., 2024) and other species of the Polygonaceae family, such as *Calligonum mongolicum* (Ullah et al., 2022). Si application significantly reduced MDA content in leaves and roots in most cases, both in control and stressed plants. This effect confirms the ability of Si to stabilize membrane structures by inhibiting lipid peroxidation (Ullah et al., 2024; Weisany et al., 2024; Morshedloo et al., 2025).

### 3.5 Total flavonoid content

**Figure 3:**
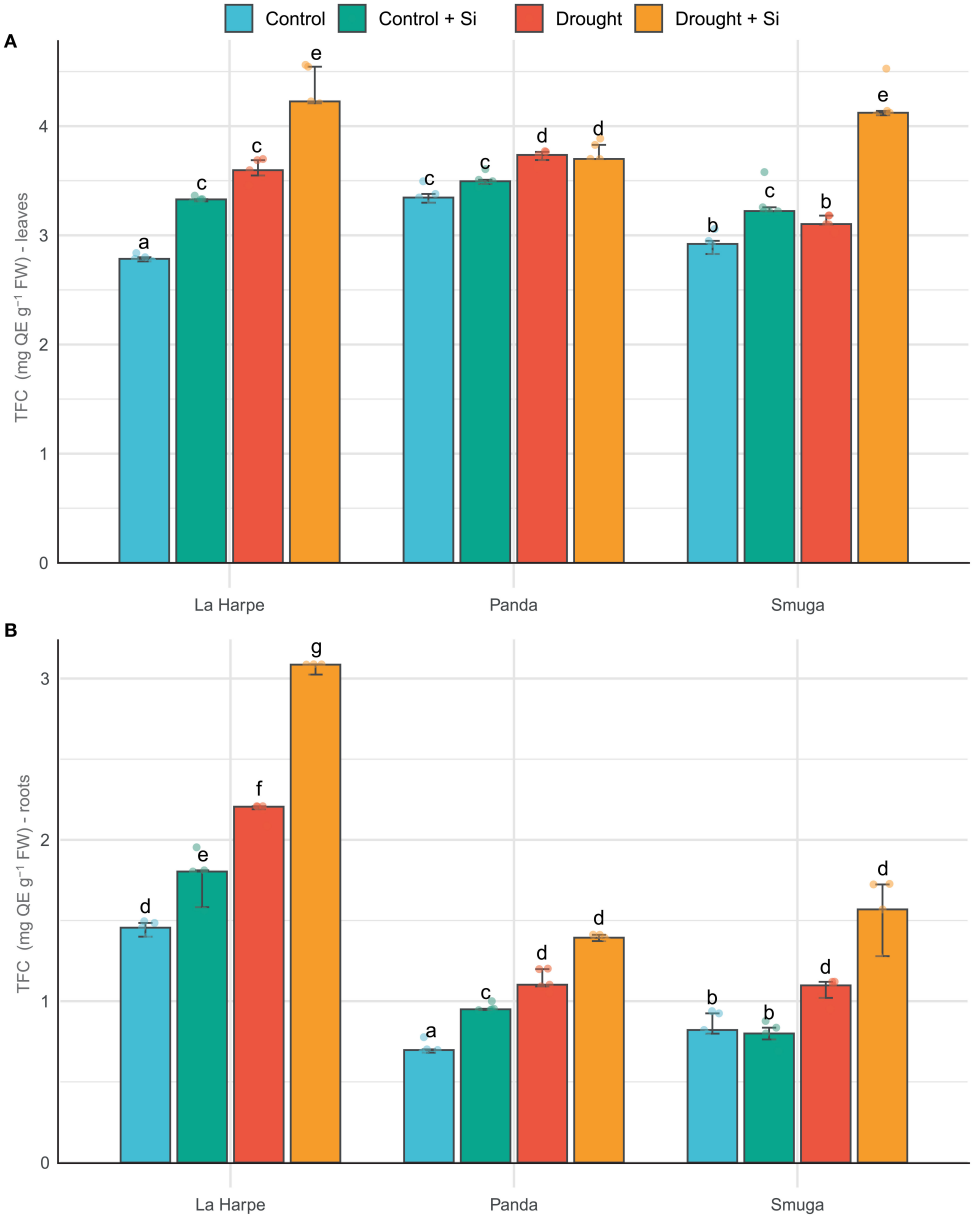
Effect of foliar application of silicon on total flavonoid content (TFC) of leaves **(A)** and roots **(B)** of different buckwheat varieties under drought conditions. Data are presented as medians ± IQR. Kruskal-Wallis ANOVA: leaves H = 55.72; *P*< 0.001; roots H = 57.01; *P*< 0.001. Different letters indicate statistically significant differences among treatments.

Flavonoids are essential antioxidants that protect plants against oxidative stress caused by such conditions as drought, excessive UV radiation, insect attack, and infections with pathogens. The increase in flavonoid content in plants under drought stress has been described in several studies (Bashir et al., 2021; Wan et al., 2023; Hossain et al., 2024) and was confirmed in our experiments. Under drought conditions, TFC was higher than Control in all varieties and organs, with or without Si (**Figure 3**). Leaves showed higher flavonoid content than roots, which could be explained by their greater UV exposure and the presence of ROS-generating reactions associated with photosynthesis. Roots synthesize flavonoids mainly for signaling to microorganisms in the rhizosphere. Foliar application of Si increased the flavonoid content even under control conditions, confirming its stimulatory effect on the production of secondary metabolites (Desoky et al., 2021; Morshedloo et al., 2025). The highest TFC values were recorded in La Harpe in the Drought + Si group, again pointing to the importance of Si in enhancing antioxidant defense systems in buckwheat varieties more sensitive to water stress.

### 3.6 Total antioxidant capacity

**Figure 4:**
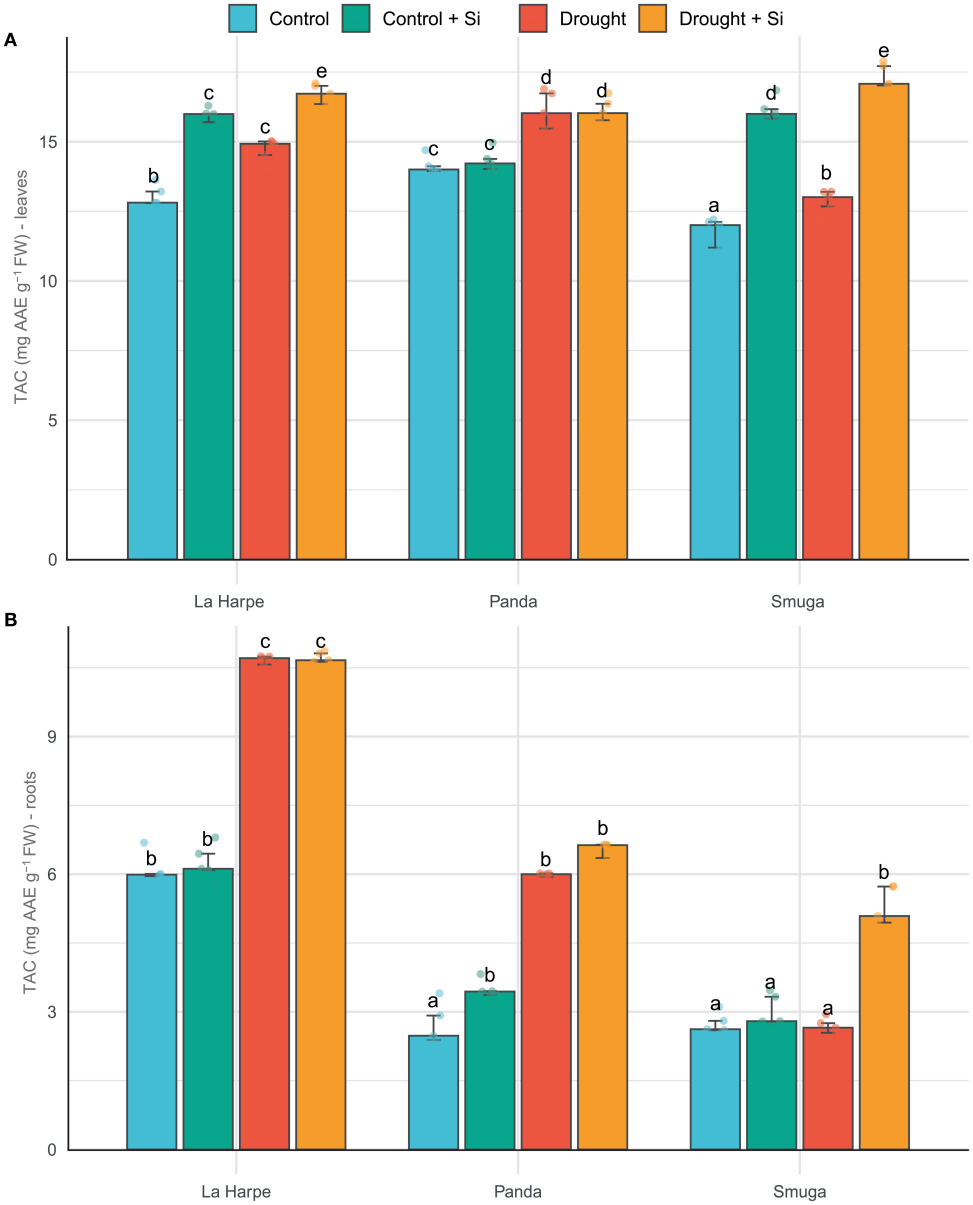
Effect of foliar application of silicon on total antioxidant capacity (TAC) of leaves **(A)** and roots **(B)** of different buckwheat varieties under drought conditions. Data are presented as medians ± IQR. Kruskal-Wallis ANOVA: leaves H = 54.37; *P*< 0.001; roots H = 54.64; *P*< 0.001. Different letters indicate statistically significant differences among treatments.

TAC expresses the ability of plants to neutralize reactive oxygen species (ROS) and protect cell structures from oxidative stress. Due to the increased need for protection against oxidative damage, TAC usually increases significantly under drought conditions (Bashir et al., 2021; Hossain et al., 2024). As shown in **Figure 4**, TAC increased under drought conditions relative to the control in all three buckwheat varieties, and TAC was higher in leaves than roots.

According to Afshari et al. (2021); Mukarram et al. (2022) and Asyhar et al. (2023), Si promotes the activity of plant antioxidant enzymes and induces the synthesis of flavonoids and phenolic compounds, thereby increasing TAC. In our results, Si application led to an increase in TAC in the leaves of all three buckwheat varieties both under drought conditions and with normal soil moisture levels. However, in Panda, the increase was slight and statistically nonsignificant (**Figure 4**). Panda did show higher basal TAC levels in leaves compared with the other varieties in both control and drought conditions. In contrast, La Harpe and Smuga showed a more pronounced response to Si application. In roots, the highest TAC content was found in La Harpe across all experimental groups, which is consistent with the TFC results.

### 3.7 Level of 5-methylcytosine

DNA methylation (5mC) is an important epigenetic mark that plays a key role in regulating gene expression. Stress signals such as drought have been shown to induce changes in DNA methylation, thereby promoting plant resistance and adaptation under adverse environmental conditions (Chang et al., 2020; Sun et al., 2022). At the molecular level, changes in 5mC levels can affect the plant’s response to abiotic stressors by suppressing or activating specific genes. Such changes in gene expression can lead to the production of secondary metabolites, which play a crucial role in signal transduction and activation of defense mechanisms under stress conditions (Anjali et al., 2023). Rao et al. (2024) reported that drought can trigger either an increase or a decrease in DNA methylation, depending on the plant species. For example, Zi et al. (2024) demonstrated a decrease in DNA methylation in *Medicago ruthenica* due to drought, while Tobiasz-Salach et al. (2023) observed an increase in methylation in corn exposed to salinity stress. In our experiments, the 5mC level in all buckwheat cultivars under drought stress was higher than in control, with the increase being statistically significant in La Harpe and Smuga. Still, nonsignificant in Panda (**Figure 1B**). Higher 5mC levels in stressed plants may be related to the suppression of genes associated with growth and the activation of genes responsible for drought tolerance.

La Harpe buckwheat appears to be the most sensitive to drought stress based on several parameters, including significantly higher levels of 5mC in leaves under drought conditions than the Panda and Smuga varieties. These results support the claim by Rao et al. (2024) that the effect of drought on DNA methylation is species-specific, and in our case, they further extend knowledge of specificity to the genotype level within a single species. Similar genotype-specific responses were also observed in a study by Zi et al. (2024), where different genotypes showed different regulation of gene expression and 5mC levels under drought conditions.

Silicon application enhances plant stress tolerance by modulating the expression of stress-related genes. For example, the effect of Si on gene expression and increased 5mC levels has been demonstrated in *Arabidopsis* under UV-B stress (Celayir et al., 2023), and in maize and wheat under salinity stress (Tobiasz-Salach et al., 2023; 2024). These molecular responses contributed to improved plant adaptation to stressful environments through modifications of biochemical and physiological traits. Similar biochemical and physiological changes were observed in our buckwheat experiment. However, under drought conditions, foliar Si application did not result in statistically significant changes in leaf 5mC levels in any of the tested cultivars (**Figure 1B**). In contrast, under control (non-stress) conditions, foliar Si application led to a statistically significant increase in 5mC levels in all varieties. This suggests that Si can induce epigenetic changes even in the absence of stress, which is consistent with the results of Yeşildirek et al. (2024), who described DNA hypermethylation after Si application in control plants of *Arabidopsis thaliana*. These results raise the possibility that pre-stress foliar application of Si may enhance plant resilience and preparedness for future drought events. This could have practical agronomic relevance, particularly in regions with frequent drought episodes during the growing season.

### 3.8 Pigment content

Chlorophyll *a* (Chl *a*) plays a key role in capturing and transferring light energy in chloroplasts, while chlorophyll *b* (Chl *b*) and carotenoids expand the spectrum of usable light in photosynthesis. Chlorophylls, particularly Chl *a*, are generally more prone to drought-induced degradation than carotenoids, which also play a protective function by stabilizing the photosynthetic apparatus and reducing oxidative stress (Saja-Garbarz et al., 2021; Thorne et al., 2021; Sattar et al., 2023).

As shown in **Table 2**, drought stress significantly reduced the levels of photosynthetically active pigments (Chl *a*, Chl *b*, total chlorophyll, and carotenoids) in all buckwheat cultivars. Foliar Si application (Control + Si) stabilized the photosynthetic apparatus and increased pigment content across all varieties. These findings are in line with studies reporting that Si, particularly in nanoparticle form, can increase pigment levels even under non-stress conditions (Akhtar and Ilyas, 2022; Al-Selwey et al., 2023; Sharf-Eldin et al., 2023). In our experiment, La Harpe and Panda responded to Si with a significant pigment increase even under regular irrigation, while Smuga showed a similar, though nonsignificant, trend—indicating genotypic variability (Azad et al., 2021; Thorne et al., 2021).

**Table 2:** Effect of foliar application of silicon on pigment content of different buckwheat varieties under drought conditions.

Variety	Treatment	*Chl a* (mg m^-2^)	*Chl b* (mg m^-2^)	*Total chlorophyll* (mg m^-2^)	*Carotenoids* (mg m^-2^)
La Harpe	Control	303.09 ± 1.90^c^	94.53 ± 1.36^d^	398.40 ± 4.25^c^	62.09 ± 1.13^b^
	Drought	227.35 ± 15.08^a^	62.59 ± 4.31^a^	292.58 ± 16.75^a^	48.50 ± 3.02^a^
	Control + Si	414.79 ± 35.77^g^	145.19 ± 25.42^f^	559.82 ± 68.49^e^	86.49 ± 9.77^e^
	Drought + Si	294.15 ± 16.00^b^	88.80 ± 5.15^d^	382.95 ± 23.54^b^	63.53 ± 1.98^c^
Panda	Control	340.88 ± 14.69^d^	98.34 ± 7.29^d^	442.69 ± 21.29^c^	71.92 ± 2.06^c^
	Drought	305.04 ± 11.61^c^	88.33 ± 0.33^c^	393.32 ± 11.93^b^	62.90 ± 2.76^b^
	Control + Si	400.85 ± 8.57^f^	125.81 ± 5.93^e^	526.66 ± 17.57^d^	80.66 ± 1.72^d^
	Drought + Si	310.18 ± 22.92^d^	90.09 ± 10.25^d^	400.27 ± 35.45^c^	63.12 ± 3.21^b^
Smuga	Control	356.39 ± 9.12^e^	112.67 ± 2.25^e^	469.06 ± 5.41^d^	74.21 ± 0.27^d^
	Drought	304.58 ± 10.28^c^	85.34 ± 3.63^b^	389.92 ± 13.90^b^	64.78 ± 2.88^c^
	Control + Si	376.64 ± 9.42^e^	128.63 ± 8.66^e^	507.61 ± 15.74^d^	82.27 ± 2.51^d^
	Drought + Si	325.74 ± 8.48^d^	92.60 ± 4.60^d^	417.73 ± 12.48^c^	68.23 ± 1.26^c^
Kruskal-Wallis ANOVA		52,46	51,94	52,24	53,12
*p*		<0.001	<0.001	<0.001	<0.001

Different letters indicate statistically significant differences among treatments (α = 0.05).

Under drought, Si-treated plants maintained higher pigment levels than those without Si application, confirming the protective role of Si observed in other crops (Maghsoudi et al., 2016; Ahsan et al., 2023; Ning et al., 2023). Drought typically leads to chlorophyll degradation, whereas Si improves water retention, RWC and osmotic potential (Ψ_s_), and stabilizes the photosynthetic apparatus (Saja-Garbarz et al., 2021; Sattar et al., 2023). Our results suggest that exogenous Si helps protect photosynthetic pigments from oxidative damage, potentially preserving photosynthetic activity under drought conditions (Al-Selwey et al., 2023; Sharf-Eldin et al., 2023). This protective effect of Si was reflected in chlorophyll levels, which increased significantly in all three cultivars. However, a statistically significant rise in carotenoids was observed only in La Harpe, which also exhibited the lowest overall pigment content under water deficit conditions, indicating its higher sensitivity compared to Panda and Smuga.

### 3.9 Maximum quantum efficiency of PSII

The maximum quantum efficiency of photosystem II (Fv/Fm) is one of the most commonly used parameters for evaluating the state of the photosynthetic apparatus through chlorophyll fluorescence. Under non-stress conditions, Fv/Fm values usually range from 0.79 to 0.85. A decrease in this value indicates damage to PSII and a reduction in photosynthetic activity, often because of stress (Maghsoudi et al., 2015; Zhang et al., 2018).

In all treatment groups in our study, Fv/Fm values ranged from 0.82 to 0.84 (**Table 3**). The differences between the groups were statistically nonsignificant, suggesting that the imposed level of drought stress did not compromise the stability of PSII. This is consistent with the literature stating that Fv/Fm remains stable until plants face severe stress (Hajizadeh et al., 2022; Kalal et al., 2022; Morshedloo et al., 2025).

**Table 3:** Effect of foliar application of silicon on Fv/Fm, *A*, *E*, *g_s_*, WUE and WUE_i_ of different buckwheat varieties under drought conditions.

Variety	Treatment	*Fv/Fm*	*E* (mmol H_2_O m^-2^ s^-1^)	*g_s_* (mol H_2_O m^-2^ s^-1^)	*A* (μmol CO_2_ m^-2^ s^-1^)	*WUE*	*WUE_i_*
La Harpe	Control	0.84 ± 0.01^a^	2.04 ± 0.74^b^	0.15 ± 0.09^b^	19.23 ± 0.65^a^	7.55 ± 2.72^a^	105.97 ± 56.53^a^
	Drought	0.83 ± 0.01^a^	0.8 ± 0.33^a^	0.05 ± 0.02^a^	4.85 ± 1.49^b^	6.10 ± 0.78^b^	110.36 ± 23.22^a^
	Control + Si	0.84 ± 0.01^a^	2.6 ± 0.32^c^	0.21 ± 0.04^c^	21.66 ± 1.75^c^	8.28 ± 2.28^a^	102.33 ± 37.28^a^
	Drought + Si	0.83 ± 0.01^a^	1.67 ± 0.37^b^	0.11 ± 0.03^b^	18.51 ± 2.11^a^	11.55 ± 0.65^a^	175.36 ± 16.36^a^
Panda	Control	0.83 ± 0.02^a^	2.14 ± 0.23^b^	0.16 ± 0.03^b^	19.86 ± 2.07^a^	8.91 ± 0.61^a^	121.23 ± 14.86^a^
	Drought	0.82 ± 0.01^a^	0.92 ± 0.36^a^	0.06 ± 0.02^a^	9.55 ± 3.87^b^	11.98 ± 2.14^a^	219.67 ± 46.94^a^
	Control + Si	0.83 ± 0.01^a^	2.19 ± 0.06^b^	0.16 ± 0.01^b^	20.99 ± 0.93^c^	9.43 ± 0.75^a^	132.43 ± 12.78^a^
	Drought + Si	0.82 ± 0.02^a^	1.48 ± 0.39^b^	0.1 ± 0.03^b^	16.10 ± 1.96^a^	11.63 ± 0.72^a^	181.11 ± 28.16^a^
Smuga	Control	0.83 ± 0.01^a^	2.24 ± 0.13^c^	0.17 ± 0.02^c^	24.20 ± 1.66^c^	10.32 ± 0.86^a^	135.71 ± 15.43^a^
	Drought	0.83 ± 0.01^a^	1.62 ± 0.84^b^	0.11 ± 0.07^b^	15.51 ± 11.5^a^	10.45 ± 1.27^a^	176.25 ± 22.50^a^
	Control + Si	0.83 ± 0.01^a^	2.2 ± 0.26^b^	0.16 ± 0.03^b^	21.20 ± 2.14^c^	9.94 ± 1.50^a^	131.24 ± 22.90^a^
	Drought + Si	0.83 ± 0.01^a^	1.42 ± 0.27^b^	0.08 ± 0.03^b^	16.61 ± 1.44^a^	11.90 ± 1.58^a^	203.13 ± 50.81^a^
Kruskal-Wallis ANOVA		20,00	49,014	49,14	46,68	30,93	37,61
*p*		0.0453	<0.001	<0.001	<0.001	0.001	<0.001

Different letters indicate statistically significant differences among treatments (α = 0.05).

However, if plants activate effective defense mechanisms, such as increased production of antioxidants (TFC and TAC under drought conditions), the adverse effects of stress on PSII can be minimized, with Fv/Fm values close to normal (Zhang et al., 2018; Mahmoud et al., 2023; Hossain et al., 2024). This is further supported by our findings, which are in agreement with earlier studies reporting stable Fv/Fm values under mild to moderate drought stress when protective responses are effectively triggered (Maghsoudi et al., 2015; Mukarram et al., 2022; Morshedloo et al., 2025).

### 3.10 Net photosynthetic rate

The net photosynthetic rate (*A*) reflects the efficiency of CO_2_ fixation by the plant during photosynthesis. Drought generally inhibits photosynthetic activity by stomatal closure, which reduces CO_2_ uptake, but can also cause damage to PSII (Hussain et al., 2019; Bashir et al., 2021). Foliar Si application can enhance *A* under drought conditions (Zhang et al., 2018; Alam et al., 2024) by increasing water availability through reduction in transpiration (Cooke and Carey, 2023), by protecting chloroplasts for more efficient light absorption (Bhardwaj and Kapoor, 2021), and by reducing oxidative stress through increased antioxidant levels (TFC, TAC) (Mukarram et al., 2023).

Our results under drought conditions showed a significant decrease in *A* in all three buckwheat varieties (**Table 3**). La Harpe and Panda, which were subjected to water deficit, showed lower *A*, *E* and *g_s_* compared with the Smuga variety, indicating their higher sensitivity to drought stress, and the greater drought resistance of Smuga buckwheat. These results are in agreement with the studies of Bashir et al. (2021) and Nyaupane et al. (2024), which also indicated that drought stress caused a decrease in *A*, *E* and *g_s_*, and that there were significant varietal differences in plant responses to drought (Hussain et al., 2019).

Si application significantly increased *A* for Control + Si and especially for Drought + Si in the La Harpe and Panda varieties. In Smuga, the effect of Si on *A* was not statistically significant. Similar varietal variations in response to Si application have been documented by Thorne et al. (2021). As demonstrated by other physiological parameters, we showed that Si application contributed to the increased drought tolerance of La Harpe and Panda. Improvement in photosynthetic activity after Si application under drought conditions was also reported by Zhang et al. (2018); Bhardwaj and Kapoor (2021) and Alam et al. (2024). In the Smuga variety, other drought tolerance mechanisms that were not activated in the more susceptible cultivars appeared to be involved. Nyaupane et al. (2024) also pointed out the potential differences in physiological mechanisms of drought resistance within a single species.

### 3.11 Transpiration rate (*E*) and stomatal conductance (*g_s_*)

The stomata regulate both CO_2_ input for photosynthesis and water vapor output during transpiration; therefore, stomatal conductance, *g_s_*, represents a plant’s efficiency in regulating gaseous fluxes between the cells and the atmosphere. Under drought conditions, there is a decrease in *g_s_* and *E* as plants wholly or partially close stomata to minimize water loss (Adjah et al., 2025). Foliar application of Si can improve leaf water balance, stabilize membranes, and decrease *g_s_* and *E* (Bhardwaj and Kapoor, 2021; Al-Selwey et al., 2023; Nazim et al., 2024).

Our results demonstrated a significant reduction in *g_s_* and *E* in all three buckwheat varieties in a drought (**Table 3**), accompanied by a decrease in *A*. The La Harpe and Panda varieties again showed greater drought sensitivity through significantly lower *g_s_*, *E* and *A* values relative to Smuga. Genotypic variation in drought tolerance mechanisms within a species has been highlighted in Shams et al. (2022) and Nyaupane et al. (2024).

Foliar application of Si significantly increased *g_s_* and *E* in La Harpe in both Control + Si and Drought + Si treatments, and in Panda with Drought + Si treatment. The beneficial effects of Si on *g_s_* and *E* in cotton plants were described by Khalequzzaman et al. (2024) under both control and stress conditions. Similar results were also reported by Bhardwaj and Kapoor (2021); Maghsoudi et al. (2016); Zhang et al. (2017) and Nazim et al. (2024). However, in the Smuga cultivar, the application of Si did not increase *g_s_* and *E*, nor did it even lead to a slight decrease. This suggests that different resistance mechanisms, such as more efficient osmotic regulation, might be involved in this buckwheat variety, as evidenced by the highest proline accumulation recorded in the Drought + Si group.

### 3.12 Water use efficiency

Instantaneous water use efficiency (WUE) is defined as the ratio of *A* to *E*, while internal water use efficiency (WUE_i_) is expressed as the ratio of *A* to *g_s_*. WUE_i_ indicates how efficiently a plant uses CO_2_ for photosynthesis at a given stomatal opening level (Zhu et al., 2021; Liang et al., 2023).

In our study, the WUE parameter was significantly lower only in La Harpe under water deficit conditions, indicating that the decrease in *A* was more pronounced than the decrease in *E*, probably due to a non-stomatal limitation of photosynthesis. In contrast, Si treatment of La Harpe in drought showed a significant increase in WUE. No statistically significant differences in WUE values were observed in the other varieties or treatment groups. Thus, the results confirmed the beneficial effect of Si on WUE only in the La Harpe plants. Similarly, Mahmoud et al. (2023) showed an increase in WUE after Si application in coriander. Meanwhile, Liang et al. (2023) concluded that genetic factors could significantly affect WUE and WUE_i_. WUE_i_ did not show significant differences among the buckwheat varieties evaluated in the different groups. However, higher WUE_i_ values were recorded under water deficit compared to the Control, which may be attributed to the drought-induced decrease in *g_s_*. Similar findings were reported by Barratt et al. (2023) for sugar beets and Han et al. (2023) for cotton. Nazim et al. (2024) also reported that plants responded to water deficit by closing stomata, thus increasing WUE_i_ as an adaptive response to limited water availability.

### 3.13 Group correlation analysis

A broad set of monitored physiological and biochemical parameters enabled the identification of traits in which buckwheat varieties differ in drought tolerance and response to foliar silicon application. Under drought stress, buckwheat plants exhibit several changes in their habitus, including reduced vegetative growth (**Supplementary Figure S1**). A group correlation analysis was performed to further evaluate different varieties’ physiological integrity and stress response. Parameters were classified into two functional categories: traits associated with physiological performance and plant vitality (“health”), and traits involved in stress response and defense mechanisms (“defense”), as defined in **Supplementary Table S1**.

The average intergroup correlation values (health–defense) are summarized in **Supplementary Table S1**. Under drought conditions, the Smuga variety exhibited the highest health–defense correlation (0.07), indicating a relatively well-preserved coordination between physiological functioning and defense activation. In contrast, Panda and La Harpe showed weaker or even negative correlations (0.02 and −0.04, respectively), suggesting impaired physiological integration under water deficit.

Following foliar Si application, health–defense correlations improved in all varieties, most notably in Smuga (Δ = +0.18), followed by Panda (Δ = +0.15) and La Harpe (Δ = +0.08). These findings suggest that silicon can improve systemic physiological coordination under drought across buckwheat varieties differing in stress sensitivity. Notably, the strongest improvement observed in Smuga indicates that even drought-tolerant varieties may significantly benefit from silicon in terms of maintaining functional integrity under stress conditions.

## 4 Conclusions

The study results showed that buckwheat’s response to water stress, including changes in physiological activity, stress responses and the involvement of defense mechanisms, is variety-specific. The Smuga variety showed the highest level of tolerance to drought, while Panda was moderately sensitive among the evaluated varieties, and La Harpe was the most sensitive to water stress. Foliar silicon application improved physiological parameters and plant resistance under drought conditions. The strongest improvement in Smuga, determined by group correlation analysis, suggests that even drought-tolerant varieties can significantly benefit from strengthening the systemic physiological integrity of plants when foliar Si is applied. This confirms the potential of Si to mitigate the negative impact of drought and highlights the importance of varietal variability in assessing plant stress tolerance mechanisms.

## Data Availability

The original contributions presented in the study are included in the article/**Supplementary Material**. Further inquiries can be directed to the corresponding author.
