# Supplementary material for: Silicon application enhances drought resilience in buckwheat: a comparative study of three varieties
Source: Front Plant Sci. 2025 Sep 23;16:1635709. doi: 10.3389/fpls.2025.1635709 (PMC12503412; doi:10.3389/fpls.2025.1635709)
Supplement: Supplementary file 1 [file DataSheet1.docx]

Supplementary Material

**Supplementary Table 1.** Group correlation analysis. Summary of average pairwise Pearson correlation coefficients between two functional groups of parameters in three buckwheat varieties under different treatment conditions.

| **Variety** | **Treatment** | **Health-Defense** | **Δ health defense** |
| --- | --- | --- | --- |
| La Harpe | Control | 0.06 | - |
| La Harpe | Control + Si | 0.08 | - |
| La Harpe | Drought | -0.04 | - |
| La Harpe | Drought + Si | 0.04 | 0.08 |
| Panda | Control | 0.18 | - |
| Panda | Control + Si | -0.02 | - |
| Panda | Drought | 0.02 | - |
| Panda | Drought + Si | 0.17 | 0.15 |
| Smuga | Control | 0.05 | - |
| Smuga | Control + Si | -0.05 | - |
| Smuga | Drought | 0.07 | - |
| Smuga | Drought + Si | 0.25 | 0.18 |

The parameters were divided into two functional groups:

1. *Health-related traits* (this functional category included traits related to the correct physiological function of plants, or rather, their health): RWC, Ψₛ, Chl *a*, Chl *b*, total chlorophyll, Fv/Fm, *A*, *E*, *g_s_*, WUE, WUE_i_,
2. *Defence-related* traits (this functional category included traits related to the response to stress, or plant defense): proline, MDA, TFC, TAC, 5mC, carotenoids.

For function groups defined in **Supplementary Table 1**, we computed the mean correlation coefficient across all trait combinations in that pair. This led to the creation of summary metrics per variety/treatment that reflect the relationship/match between proper plant physiological function (health) on the one hand and stress level and defense response (defense) on the other. The intensity of the green color highlights the degree of improvement in the correlation values between Drought and Drought + Si treatments. All values are rounded to two decimal places.


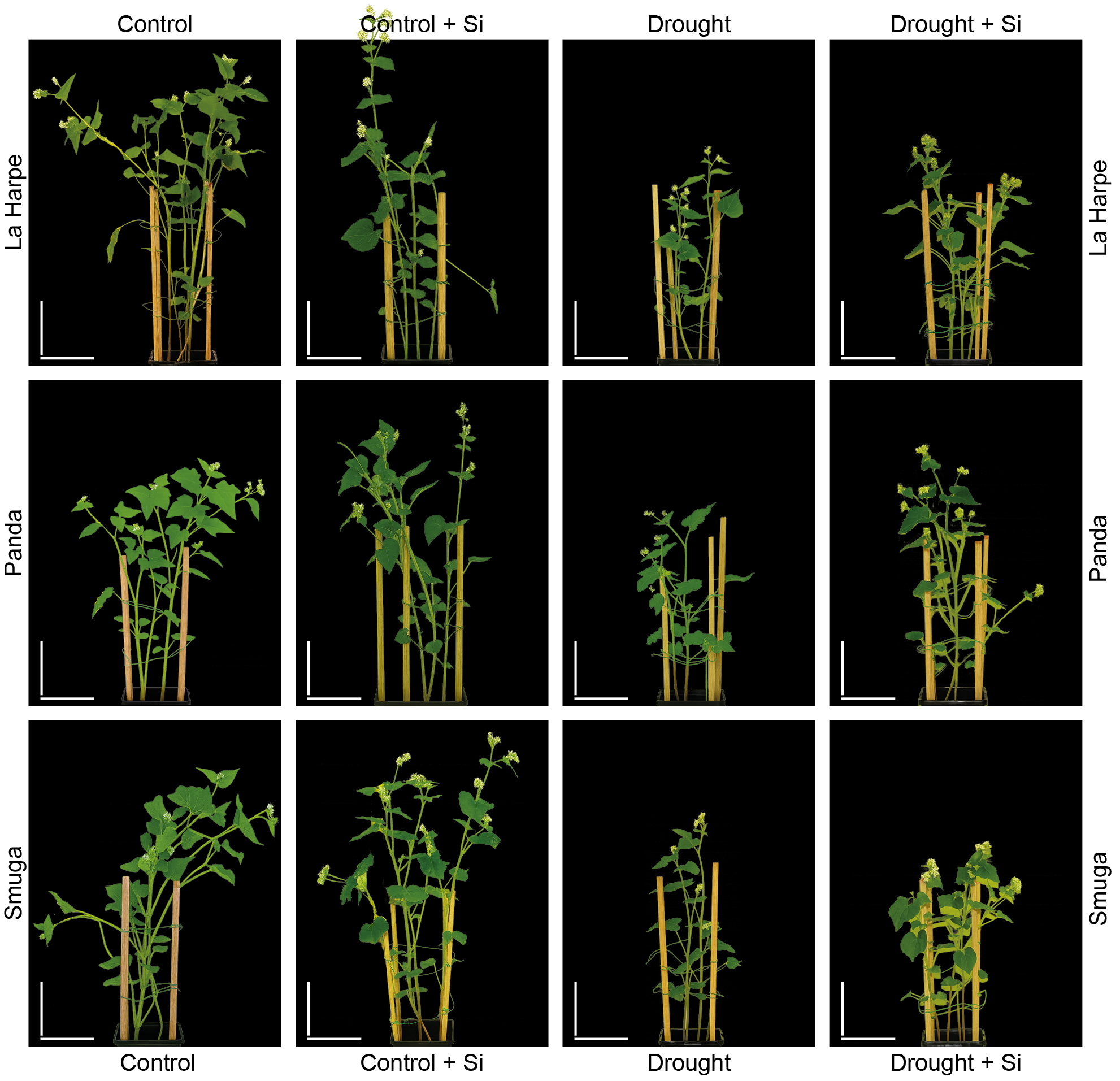


**Supplementary Figure 1.** Under drought stress, buckwheat plants exhibit several changes in their habitus, including reduced vegetative growth (bar = 10 cm).
